# The Effect of the Variation in Al_2_O_3_ and SrO Content on the Structure, Sintering Behavior, and Properties of SrO, BaO, ZnO, MgO-B_2_O_3_-Al_2_O_3_-SiO_2_ Glass-Ceramics for Use in Al_2_O_3_ Ceramic LTCC Applications

**DOI:** 10.3390/ma18194510

**Published:** 2025-09-28

**Authors:** Junlin Xie, Ningning Li, Pengkai Shang, Zijun He, Lei Zhao, Feng He

**Affiliations:** 1 School of Materials Science and Engineering, Beijing University of Technology, Beijing 100124, China; xjl09532@bjut.edu.cn (J.X.); ning0526@sina.cn (N.L.); pengkai_sh2021@126.com (P.S.); 2 Department of Functional Material, Central Iron and Steel Research Institute Co., Ltd., Beijing 100081, China; doublehzj@126.com (Z.H.); zl3320@163.com (L.Z.)

**Keywords:** glass-ceramics, Al_2_O_3_ ceramic substrate, structure, sintering behavior, sintered interface

## Abstract

A systematic investigation was conducted into the effects of varying Al_2_O_3_ and SrO contents on the structure, sintering kinetics, crystallization patterns, and properties of the SrO-BaO-ZnO-MgO (RO)-B_2_O_3_-Al_2_O_3_-SiO_2_ (RBAS) system. This glass-ceramic demonstrates effective applicability for low-temperature co-firing of alumina ceramics. Increasing Al_2_O_3_ content densified the glass network and reduced crystallinity, thereby promoting sintering densification. It led to improved acid resistance and a lower coefficient of thermal expansion (CTE). The composition with 17.50 mol% Al_2_O_3_ sintered at 800 °C exhibited optimal properties: a well-matched CTE of 7.45 × 10^−6^ K^−1^, a high flexural strength of 130.58 MPa, and excellent chemical stability. Furthermore, it demonstrated excellent compatibility when co-sintered with an Al_2_O_3_ substrate.

## 1. Introduction

Since the dawn of the 21st century, the rapid advancement of the modern electronics industry has placed increasingly stringent demands upon the preparation of electronic materials [1,2,3,4,5]. Low-temperature co-fired ceramic (LTCC) technology is characterized by low-temperature sintering, a high degree of miniaturization, and the highest level of reliability, enabling high-density encapsulation of complex circuits and passive components, which helps to achieve miniaturization and high integration of electronic devices [6,7]. LTCC materials refer to a complete material system, which consists mainly of a ceramic substrate, a conductive phase, and a sealing material. The sealing materials are glass and glass-ceramics with a sintering temperature below 1000 °C. Mechanically supported, high-density encapsulated glass or glass-ceramics with excellent electrical insulation, hermetic sealing, and environmental protection properties play a vital role in the LTCC process [8,9,10,11,12]. The components and proportions of glass directly affect the performance of LTCCs, including electrical properties, quality, bending strength, sintering shrinkage, and other key indicators [13,14].

Al_2_O_3_ ceramics have high mechanical properties, excellent chemical stability, excellent dielectric properties, and moderate CTE (7.5–8.0 × 10^−6^ K^−1^) [15]. They are widely used as substrate materials in the field of microelectronics and integrated circuits, such as multilayer ceramic capacitors [16,17] and semiconductor substrates [18,19,20]. For example, in preparing multilayer ceramic capacitors using LTCC technology, it is necessary to uniformly sinter the conductive phase on an Al_2_O_3_ ceramic substrate through a sealing glass. However, if the CTE of the glass exhibits a significant mismatch with that of the Al_2_O_3_ ceramic substrate, peeling or cracking may occur at the bonding interface, resulting in device failure. Therefore, it is particularly important to develop a sealing glass that is compatible with the CTE of the Al_2_O_3_ ceramic.

Commercial LTCC materials are primarily categorized into two types: glass/ceramic systems and glass-ceramic/ceramic systems. A variety of basic glasses have been researched in response to the diverse application scenarios of LTCC. The basic glass systems that have been investigated mainly include CaO-B_2_O_3_-SiO_2_ (CBS) glass [6], MgO-B_2_O_3_-SiO_2_ (MBS) glass [7], ZnO-B_2_O_3_-SiO_2_ (ZBS) glass [21], Li_2_O-B_2_O_3_-Al_2_O_3_ (LBA) glass [22], and Li_2_O-Al_2_O_3_-SiO_2_(LAS) glass [23], RO-TiO_2_-B_2_O_3_-SiO_2_ (RTBS) glass [24]. Each basic glass has its own characteristics corresponding to its sintered base materials. Based on the fundamental properties of Al_2_O_3_ ceramics, RBAS base glass systems were designed in this work to achieve greater compatibility between the expansion coefficients and compositions of the sealing glass and Al_2_O_3_ ceramics, whilst enabling both materials to be sintered at 800 °C. In the basic glass component, Al_2_O_3_ is intentionally added to increase the adaptability and compatibility of the two materials in terms of composition.

The research on the RBAS glass system is mainly reflected in solid oxide fuel cell (SOFC) systems, and there is not much research on the application of the RBAS glass system in LTCC. Zhigachev et al. [18] studied BaO-CaO-SiO_2_-Al_2_O_3_-B_2_O_3_ sealed glasses for SOFC systems. By adjusting the SiO_2_ and B_2_O_3_ contents, sealing glasses with CETs in the range of 10.0–11.0 × 10^−6^ K^−1^ were prepared. Li et al. [25] investigated the structure and properties of BaO-Al_2_O_3_-B_2_O_3_-SiO_2_ sealing glass-ceramics. By varying the content of SiO_2_, the CTE range of the glass-ceramics was tuned from 9.77 × 10^−6^ K^−1^ to 13.37 × 10^−6^ K^−1^. BaO-ZnO-SiO_2_-B_2_O_3_ sealing glass-ceramics for SOFC with a CTE of 5.5 × 10^−6^ K^−1^ were reported by Kingnoi et al. [26]. From the above works, it can be seen that researchers are very concerned about the CTE matching between materials for device sealing. However, there are few reports on sealing glass for Al_2_O_3_ ceramic substrate sealing, and RBAS glass-ceramic is a very promising sealing material for Al_2_O_3_ ceramic substrate sealing. It is necessary to study its structure and properties to prepare RBAS glass-ceramics that are compatible with Al_2_O_3_ ceramic substrates.

For aluminosilicate glasses, the content of Al_2_O_3_ plays a crucial role in regulating their structure [27,28], glass transition temperature [29], crystalline behavior [29], CTE [30], and chemical resistance [31,32]. Herein, the unique contribution of this study lies in the systematic investigation of the structure, sintering behavior, crystallographic characteristics, and properties of a series of RBAS glass-ceramics with varying compositions, achieved by substituting SrO with Al_2_O_3_. The glass-ceramics matching the Al_2_O_3_ ceramic CTE were obtained.

## 2. Materials and Methods

### 2.1. Component Design and Sample Preparation

The chemical compositions of the RBAS glass with different Al_2_O_3_ and SrO content are summarized in Table 1. The raw materials employed comprised SiO_2_ (Sinopharm, Shanghai, China, 99%), H_3_BO_3_ (Sinopharm, 99.1%), Al_2_O_3_ (Sinopharm, 98.5%), BaCO_3_ (Sinopharm, 98.5%), SrCO_3_ (SCR, Beijing, China, 99.1%), ZnO (Sinopharm, 99.3%), TiO_2_ (Rhawn, Shanghai, China, 99.3%), MgO (SCR, 99.5%), and Na_2_CO_3_ (Sinopharm, 99.5%). After weighing and mixing, place approximately 300 g of thoroughly blended sample into a platinum–rhodium crucible. Melt in a muffle furnace at 1450 °C for 2 h to form a uniform melt. After removing the molten glass, it is immediately poured into deionized water, producing glass particles measuring 1–3 mm in diameter. The glass cullet was dried in an oven at 80 °C for 24 h, then ground in a ball mill at 150 r/min for 8 h. The resulting glass powder was sieved through a 200-mesh screen. The glass powder and 5 wt% polyvinyl alcohol solution were mixed and ground evenly, and the sintered billet was obtained by holding the pressure for 2 min under the pressure of 20 MPa. The obtained product is dried at 100 °C for 6 h, then degreased in a muffle furnace at 350 °C under air conditions for 3 h. The degreased product continues to be heated from 350 °C to 800 or 850 °C at a rate of 30 °C/min within the muffle furnace, kept at this temperature for 10 min, then cooled at a rate of 3 °C/min to ambient temperature. The resulting RBAS glass-ceramics were used for structural and various performance tests of RBAS glasses.

Mix glass powder with a solution of rosin and polyvinyl alcohol to create a slurry, then print the slurry on the surface of Al_2_O_3_ ceramic. The obtained samples are dried at 100 °C for 6 h, and then debound in a muffle furnace at 300 °C for 3 h under air conditions. After the debinding process is complete, the temperature is heated to 800 °C at a rate of 30 °C/min and maintained for 10 min to obtain LTCC samples. After cooling, the samples were cut using wire cutting to obtain cross-sections for SEM-EDS testing.

As the water quenching method was employed to prepare glass sample pieces, constituents within the glasses may have been dissolved by the deionized water. Consequently, Inductively Coupled Plasma Mass Spectrometry (ICP-MS) was utilized to analyze the composition of the RBAS glasses, thereby investigating alterations in their constituent elements. Table 2 presents the ICP-MS test results. It can be observed that the glasses compositions underwent slight changes following water quenching. The results indicate that measured chemical compositions exhibit both increases and decreases relative to the design compositions. Indeed, the measured alkali metal oxide content in RBAS glasses samples is consistently lower than the design value, potentially attributable to the dissolution of Na^+^ into water during quenching. For the prepared samples, the same quenching method may result in all actual glass samples having Na_2_O content below the design value. The measured Al_2_O_3_ content in the glasses generally exceeded its design value. The phenomenon is likely closely related to the use of Al_2_O_3_ balls for grinding the glasses, where trace amounts of Al_2_O_3_ balls were ground into the glasses’ compositions.

### 2.2. Structure and Performance Testing

Glass samples were ground and sieved through a 200-mesh sieve. Precise weights of 0.2 g were taken and subjected to microwave digestion in a polytetrafluoroethylene (PTFE) vessel using grade-reagent (GR) hydrofluoric acid (HF). The chemical composition of the digested solution was analyzed by ICP-MS using a PerkinElmer NexION 300X (PerkinElmer, Waltham, MA, USA). The characteristic temperatures of the base glass were determined using differential scanning calorimetry (DSC). The instrument utilized was a Netzsch STA449F3 (Nerz Instruments Manufacturing GmbH, Selb, Germany), with the test atmosphere being air. The temperature range spanned from room temperature to 1100 °C, ramping up at a heating rate of 10 °C/min. The vibrational modes within the glass were analyzed using Fourier Transform Infrared Spectroscopy (FTIR) with a Thermo Fisher Scientific Nicolet 6700 instrument (Thermo Fisher Scientific Instruments Limited, Waltham, MA, USA). Prior to testing, the powdered sample was uniformly ground to a particle size of <75 μm. Glass powder and KBr were weighed separately and mixed in a 1:100 ratio. The mixture was then pressed into a thin pellet and inserted into the instrument. The test wavenumber range was 400 cm^−1^ to 1600 cm^−1^, with 64 scans recorded. The crystalline phases precipitated in the glass specimens were determined using X-ray diffraction (XRD). The testing apparatus employed was a Bruker D8 Advance (Bruker AXS GmbH, Billerica, MA, USA). Measurements were carried out at ambient temperature (25 °C) with a step size of 0.02°, 2θ angle range of 10° to 70°, and Cu Kα radiation (λ = 0.154 nm) at 40 kV and 30 mA. The XRD pattern acquired through testing was analyzed using Jade software (MDI Jade 9), and thus the crystal type became determinable. XRD data analysis utilizes the powder diffraction file (PDF-2004). The vibrational modes of glass exhibit Raman scattering effects. Spectral analysis employed the LABHRev-UV instrument (Horiba, Japan), with the sample positioned on a microscope slide under the objective lens. Following focusing, photon information was collected. The testing range spanned from 200 cm^−1^ to 2000 cm^−1^. An 1800 gr/mm diffraction grating and a 100-times objective lens were employed, with a laser power of 30 mW. An argon ion laser (excitation wavelength 532 nm) served as the pump source. Spectral resolution was approximately 1–2 cm^−1^. Origin software (Origin 2024) was employed to fit the Raman shifts of each vibration mode using Gaussian functions, thereby determining the positions of overlapping spectra and ultimately yielding the deconvoluted results. Field emission scanning electron microscopy (FE-SEM), specifically the Zeiss Ultra Plus instrument from Oberkochen, Germany, was employed to investigate the micro-morphological characteristics of the materials. Bulk samples underwent surface polishing followed by etching in a 5 v% hydrofluoric acid solution at room temperature for 40 s, after which they were rinsed in distilled water within an ultrasonic cleaner for 5 min. The etched samples obtained required coating with a platinum film prior to FE-SEM analysis.

The sintering shrinkage curves were characterized utilizing a high-temperature microscope (HM867, TA Instruments, New Castle, DE, USA). In the course of the experiment, the base glass powder was thoroughly blended with a specific volume of deionized water, then compressed into a cylinder of uniform shape. The resulting specimen was heated from ambient temperature to 900 °C at a heating rate of 10 °C/min, allowing for the capture of side-view images of the sample throughout the heating process as well as the generation of the sintering shrinkage curve. This experiment employs the displacement method, utilizing Archimedes’ principle of buoyancy to calculate the bulk density of sintered microcrystalline glass. This parameter represents the mass of material per unit volume. The calculation formula is illustrated in Equation (1):

(1) $$\rho \;=\;\frac{m_{1}}{m_{1}-m_{2}}\rho_{water}$$

where *ρ* is the bulk density (g/cm^3^) of the test sample, *m*_1_ is the mass of the microcrystalline glass in air (g), *m*_2_ is the mass of the test sample in water (g), and *ρ_water_* is the density of water (g/cm^3^).

The CTE values of the materials were determined using a thermal expansion coefficient tester (Netzsch DIL402SE, Selb, Germany). Specimen dimensions were 25 mm × 4 mm × 4 mm, and the measurement was carried out in an air atmosphere. The heating process was controlled at a rate of 10 °C/min, with the temperature ranging from ambient temperature to 750 °C. The three-point bending method was employed to assess the flexural strength of specimens using a universal testing machine (Shimadzu, Kyoto, Japan; AGIC50 kN). The specimens prepared for this test were sized at 50 mm × 4 mm × 4 mm, with a test span of 25 mm and a loading rate maintained at 9.8 ± 0.1 N/s. The sintered samples cut into 12 mm × 12 mm × 12 mm were cleaned, dried, and weighed, then soaked in 10% by weight HCl solution for 0.5 h at room temperature, washed again, dried, and weighed, and the acid resistance was calculated by calculating the rate of mass loss as shown in Equation (2).

(2) $$\mu =\frac{m_{1}-m_{2}}{m_{1}}\times 100\%,$$

where *μ* denotes the rate of mass loss after acid attack, *m*_1_, *m*_2_ represent the mass of the sample before and after acid attack (g).

## 3. Results and Discussions

### 3.1. Structural Analysis of Basic Glasses

**Figure 1:**
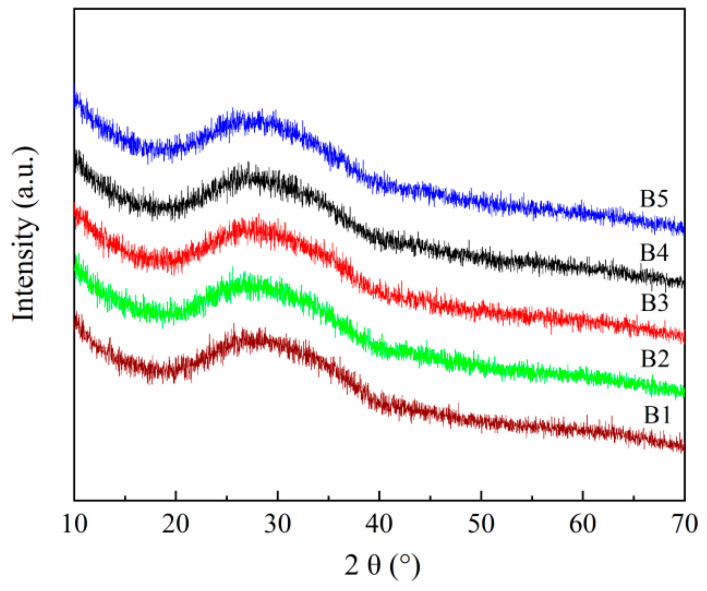
XRD patterns of the RBAS basic glasses.

Figure 1 shows the XRD spectra of the basic glass samples prepared in the experiment. It can be observed that all glass samples exhibit broad diffuse peaks within the 20° to 40° range. This characteristic serves as a key criterion for identifying the material as amorphous glass.

**Figure 2:**
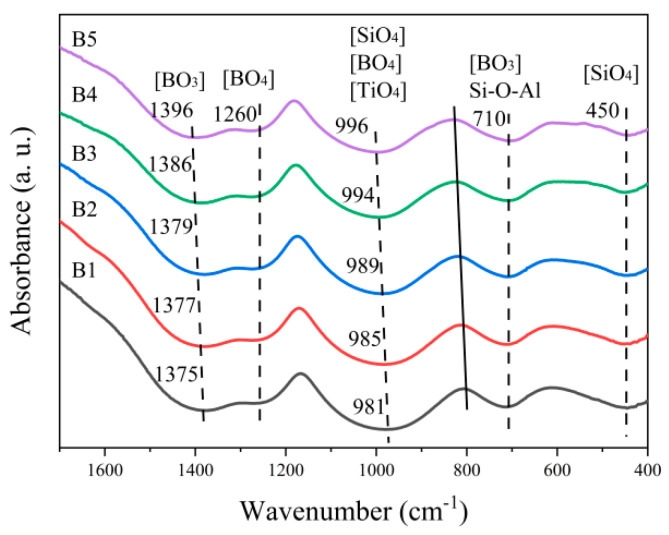
FTIR spectra of the RBAS basic glasses.

Figure 2 depicts the FTIR spectra of the RBAS basic glasses and Table 3 lists the corresponding characteristic vibrations of the absorption bands. As illustrated in Figure 2, a shoulder peak appearing within the wavenumber range of 710–800 cm^−1^ may be attributed to the stretching vibrational mode of the Si–O–Al bond. The augmentation of Al_2_O_3_ content led to a broadening of the shoulder peak, which suggests that the quantity of Si–O–Al linkages formed between [SiO_4_] and [AlO_4_] units has increased. The enhancement of the Si–O–Al structure proves that Al_2_O_3_ can stimulate the formation of hybrid networks of [SiO_4_] and [AlO_4_]. The peak of the absorption band located in the 800–1200 cm^−1^ range exhibited a shift toward a higher wavenumber, while its intensity gradually increased. This spectroscopic behavior implies that in the RBAS glass system, the quantity of bridging oxygen has increased, consequently resulting in an enhanced degree of polymerization within the glass network structure. This was partly attributable to Al_2_O_3,_ which participates in the glass network and connects with [SiO_4_] to form the network, thus making the glass network structure dense. On the other hand, the free oxygen introduced by SrO causes Si–O–Si fracture, and the reduction in SrO content weakens the network fracture [33]. The absorption band proximate to 1380 cm^−1^ exhibited a shift from the lower wave number towards the higher one, signifying an augmentation of [BO_3_] and a diminution of [BO_4_] within the glass network. This phenomenon could be ascribed to the increment in Al_2_O_3_ content. Specifically, the rise in Al_2_O_3_ content engendered a reduction in the free oxygen content and a gradual decline in the [BO_4_] content.

**Figure 3:**
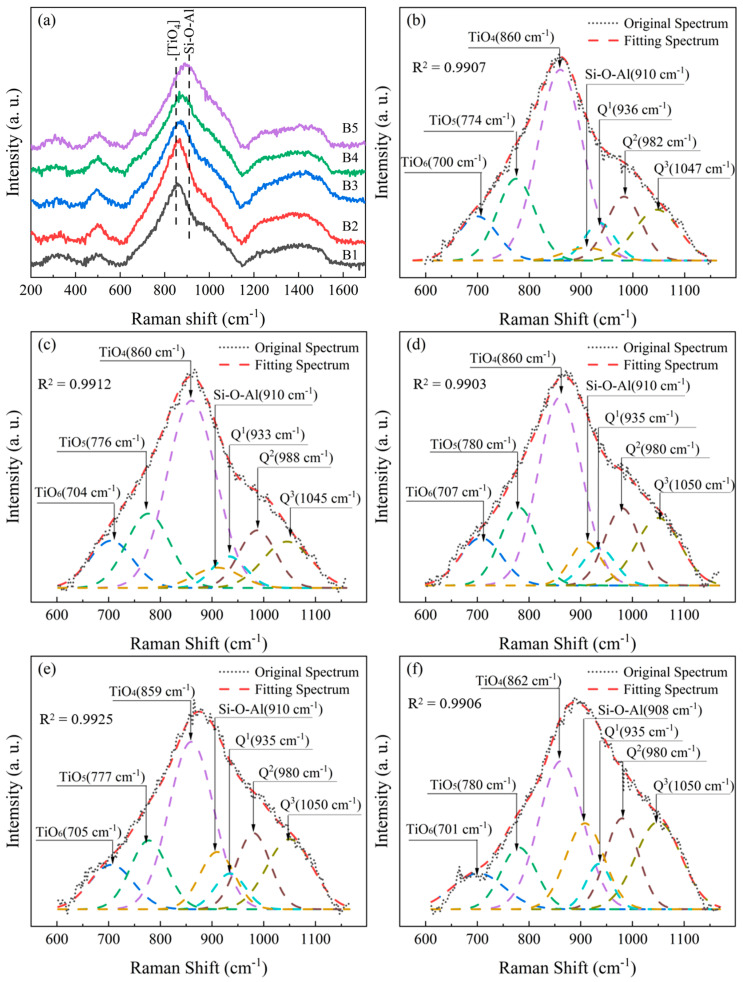
Raman spectra (**a**) and deconvolved results (**b**–**f**) of the RBAS basic glasses (**b**–**f**: B1–B5).

The Raman spectra of RBAS basic glasses with different Al_2_O_3_ contents are plotted in Figure 3a, and the distribution of Raman spectra is given in Table 4. The peak observed at 310 cm^−1^ was ascribed to cationic vibration. In the spectral range of 600–1100 cm^−1^, the peaks corresponded to vibrations associated with [TiO_n_], Si–O–Al, and Q^n^ (where Q^n^ represents the structural unit of the silica network, with Q denoting the silicon tetrahedron and n signifying the number of oxygen bridges contained within each tetrahedron). Meanwhile, the peaks falling within the 1100–1600 cm^−1^ interval were attributed to B–O vibration. With the escalating content of Al_2_O_3_, the vibration peak intensity of [TiO_6_] exhibited a progressive augmentation. Concurrently, the Si–O–Al structure also witnessed a gradual increment, which led to the broadening of the Raman spectrum peak within the range of 600–1150 cm^−1^ and a conspicuous shift of the peak towards the higher wave number.

Gaussian functions were used to deconvolute overlapping peaks in the Raman spectra. The R^2^ value is employed to evaluate model fit, serving as an indicator of goodness-of-fit. The closer the R^2^ value approaches 1, the higher the degree of correspondence between the model and the data. The R^2^ values in Figure 3 are all >0.99, indicating minimal fitting error. Detailed information on the fitting results is shown in Figure 3b–f, while the peak area ratios reflecting different structures are listed in Table 5. As revealed by the fitting results, in tandem with the augmentation of Al_2_O_3_ content, the relative abundances of [TiO_6_] and Q^3^ exhibited a progressive increment, whereas the relative abundances of [TiO_4_], Q^1^, and Q^2^ manifested a gradual decline. At the same time, the concentration of free oxygen diminishes, thereby triggering the conversion from [TiO_4_] to [TiO_6_]. Furthermore, the disconnection of the glass network was suppressed, and the proportion of Q^3^ showed a gradual increase, while the proportions of Q^1^ and Q^2^ showed a gradual decrease. As the Al_2_O_3_ content increases, the intensity of the Raman peak corresponding to the Si–O–Al structure increases progressively, suggesting that Al_2_O_3_ is likely to facilitate the formation of a mixed network consisting of [SiO_4_] and [AlO_4_] structures.

**Figure 4:**
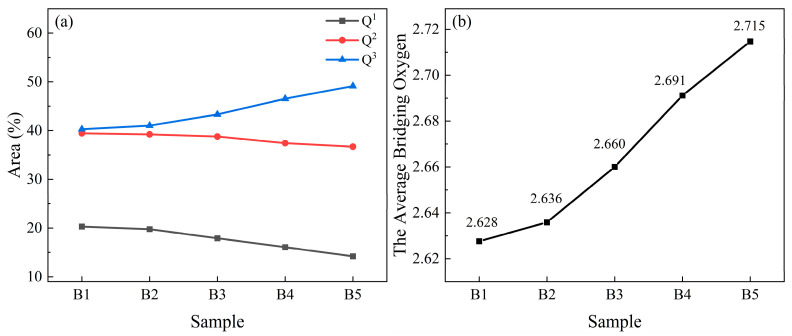
Area fraction of Q^n^ (**a**) and BO numbers level (**b**) of the RBAS basic glasses (The uncertainty of area fraction of Q^n^ and BO number measurements is below 1%).

The mole fraction of Q^n^ (n = 1, 2, and 3) is calculated as Equation (3):

(3) $$X^{n}=\frac{A_{n}}{S_{n}\sum \left(A_{n}/S_{n}\right)}$$

where X^n^ is the mole fraction of Q^n^, Sn (0.514, 0.242 and 0.09 for S1, S2, and S3, respectively [51]) is the Raman scattering coefficient of Q^n^, and An is the area fraction of Q^n^. The area fraction of Q^n^ is illustrated in Figure 4a. The average bridging oxygen number of Si is calculated through Equation (4) [52]:

(4) $$\mathrm{BO}\;\mathrm{numbers}=\sum nX_{n}$$

The average BO number of RBAS glasses with different Al_2_O_3_ content is calculated as shown in Figure 4b. As the content of Al_2_O_3_ rises, the average number of bridging oxygen atoms exhibits an upward trend, which implies that the degree of polymerization of the silicon network within the glasses has enhanced. Figure 4 is derived from Figure 3, and thus, R^2^ indirectly reflects the error in Figure 4.

### 3.2. Differential Thermal Analyses

**Figure 5:**
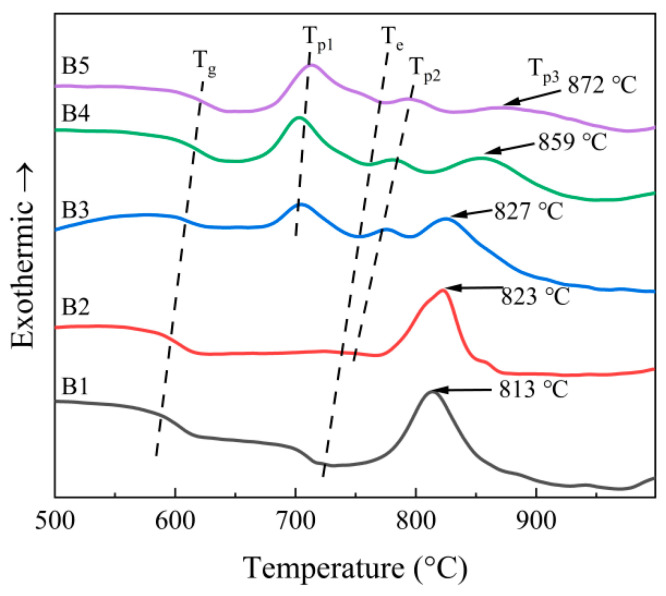
DSC curves of the basic glasses with the heating rate of 10 °C/min.

Figure 5 shows the DSC curve of RBAS basic glasses. The characteristic temperature values of the DSC curves are revealed in Table 6. T_g_ denotes the glass transition temperature, and T_e_ corresponds to the temperature at which the liquid phase appears during the sintering process of glass particles. It can be observed that the T_g_ and T_e_ tend to increase gradually with the increase of Al_2_O_3_ content. The exothermic peak in DSC represents the crystallization peak, and its associated temperature is the crystallization temperature (T_p_). Figure 5 presents three crystallization temperatures (T_p1_, T_p2_, and T_p3_), of which T_p1_ occurs in B3–B5 samples and *T*_p2_ in B2-B5 samples. Since the sintering endothermic peak of B2 and the exothermic peak of T_p2_ have similar temperature values, T_e_ and T_p2_ are not obvious in B2, and the peaks and peak widths increase slightly with the growth of Al_2_O_3_ content. With the increasing content of Al_2_O_3_, the peak intensity of the T_p3_ peak diminished and the peak temperature increased, accompanied by a broadening of the peak. This indicates that the precipitation of the crystalline phase corresponding to T_p3_ might be restrained as the Al_2_O_3_ content grows.

### 3.3. Sintering Behavior of the Basic Glasses

**Figure 6:**
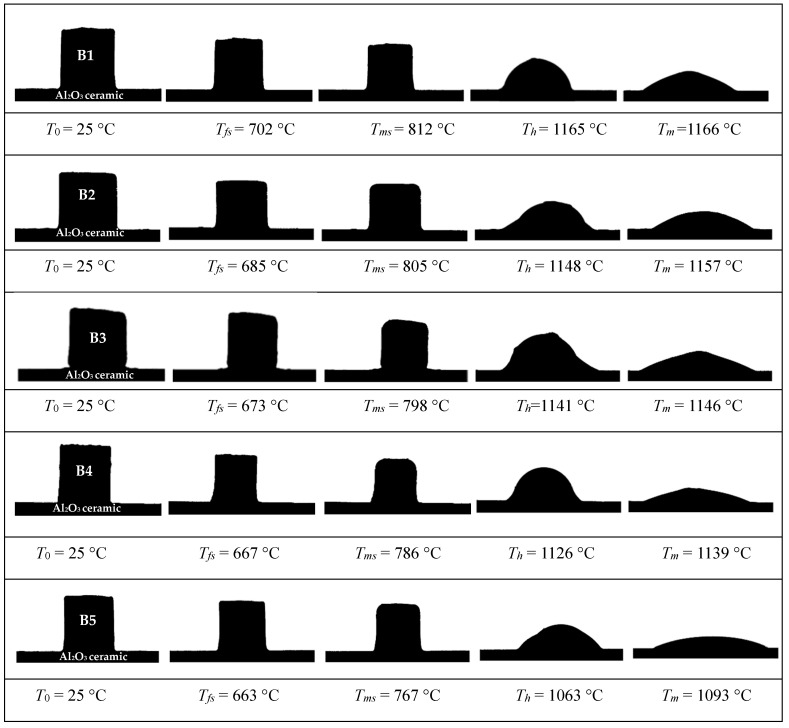
HTM images of the RBAS glasses sintered at 10 °C/min.

**Figure 7:**
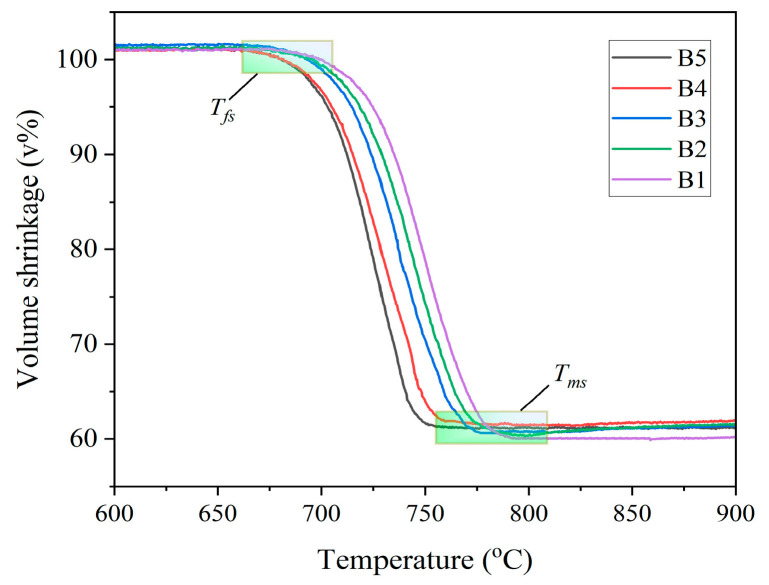
Sintering volume shrinkage curves of the glasses sintering at 10 °C/min.

Figure 6 and Figure 7 present the high-temperature microscope (HTM) images and shrinkage curves of the glasses at a heating rate of 10 °C/min, respectively. As observed from Figure 6, the glass volume experiences a slight increase within the temperature range of 25 °C to 650 °C. Sintering initiates around 675–700 °C, accompanied by volume contraction. The maximum shrinkage occurs between approximately 756 °C and 809 °C; the glass volume shrinkage reaches its peak, corresponding to the sintering densification point of the glass powder. Moreover, as the Al_2_O_3_ content rises, the sintering shrinkage diagram of RBAS glasses evidently reveals that the temperature interval during which the sample volume stays stable is notably reduced. This phenomenon is probably related to the crystallization of the glass during sintering. Higher Al_2_O_3_ content improves the network connectivity of the glass, which inhibits crystallization.

As depicted in Figure 7, an increase in Al_2_O_3_ content leads to a decrease in both the shrinkage initiation temperature (T_fs_) and the maximum shrinkage temperature (T_ms_) of the glass. This indicates that as the Al_2_O_3_ content increases, the sintering properties of RBAS glass are optimized. The hemispherical temperature (T_h_) and flow temperature (T_m_) of glass also decrease with the increase of Al_2_O_3_ content. The reason is that XRD analysis shows that crystallization occurs during the sintering process of glass powder. Crystallization fixes atoms and hinders their migration, which is unfavorable to the overall sintering procedure. A comprehensive analysis indicates that higher Al_2_O_3_ content exerts an inhibitory effect on glass crystallization. This, in turn, helps lower the sintering temperature and enhances the sintering performance of RBAS glass. The sintering of B1, B2, B3, and B4 glasses can all be implemented at temperatures below 800 °C.

### 3.4. Crystal Phase Analysis

**Figure 8:**
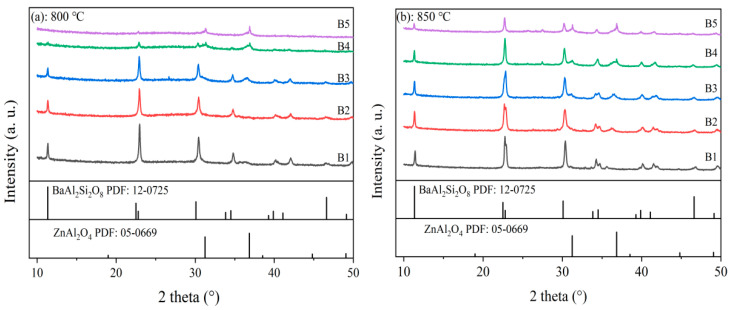
XRD patterns of the RBAS glasses sintered at 800 °C and 850 °C.

Figure 8 displays the XRD patterns of RBAS glass after sintering at 800 °C (a) and 850 °C (b). As shown in Figure 1, the base glass is amorphous, while Figure 8 indicates that the sintered glass samples exhibit distinct diffraction peaks. This suggests that microcrystalline phases have precipitated out of the glass phase. Regardless of whether the glass was sintered at 800 °C or 850 °C, the intensity of the diffraction peaks decreases with increasing Al_2_O_3_ content. Under both sintering temperature conditions, the primary crystalline phase in all five groups of samples was BaAl_2_Si_2_O_8_ crystals, while some samples exhibited the secondary crystalline phase ZnAl_2_O_4_. The exothermic peak associated with BaAl_2_Si_2_O_8_ crystals corresponds to Tp3 in the DSC curves. Specifically, at 800 °C sintering, the XRD patterns of samples B3, B4, and B5 showed diffraction peaks corresponding to those on the ZnAl_2_O_4_ crystal standard card. Similarly, at 850 °C sintering, the XRD patterns of samples B2, B3, B4, and B5 also exhibited such diffraction peaks. This phenomenon indicates that as the Al_2_O_3_ content increases, a small amount of ZnAl_2_O_4_ crystals precipitate, and the intensity of the main diffraction peaks of ZnAl_2_O_4_ crystals shows an upward trend, indicating that the precipitation of ZnAl_2_O_4_ crystals is promoted. Conversely, the precipitation process of BaAl_2_Si_2_O_8_ crystals is inhibited. Additionally, it was observed that an increase in sintering temperature promotes the crystallization process. However, it should be noted that as the Al_2_O_3_ content increases, the degree of polymerization of the RBAS glass network structure is enhanced. This is because tetracoordinate Al_2_O_3_ is incorporated into the silica–oxygen network, forming Si–O–Al bonds between [SiO_4_] and [AlO_4_] groups, thereby inhibiting the formation of BaAl_2_Si_2_O_8_ crystals [53].

**Figure 9:**
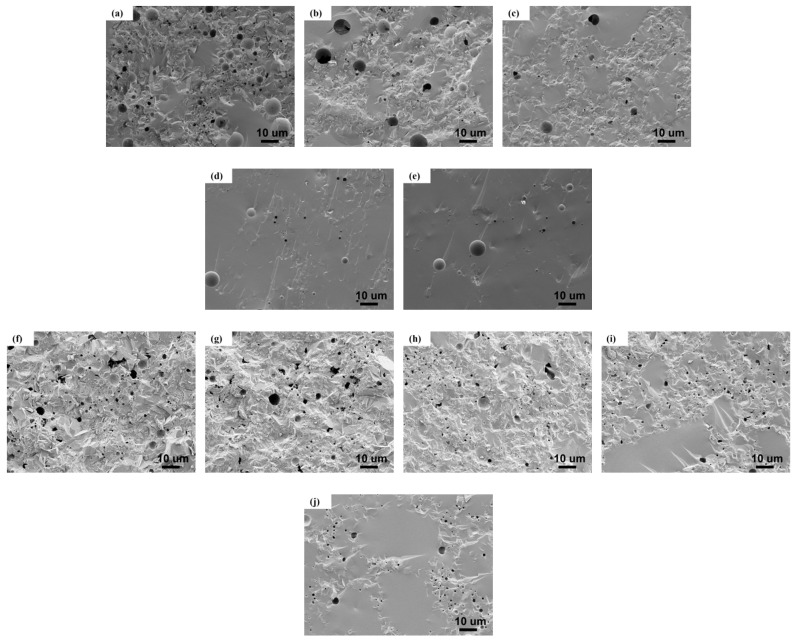
SEM images of cross-section of RBAS glass-ceramics sintered at 800 °C and 850 °C; (**a**–**e**): 800 °C B1–B5, (**f**–**j**): 850 °C B1–B5.

SEM images of cross-section of RBAS basic glass sintered at 800 and 850 °C are shown in Figure 9. With the increase of Al_2_O_3_ content, the sintering compactness of the glass increases. On the one hand, this is due to the increased polymerization of the glass network. On the other hand, as the crystallinity decreases, the larger pores produced during the sintering process gradually become smaller and their number gradually decreases, resulting in the appearance of a dense glass phase over a large area of the B4 and B5 sealed glass segments. This indicated that as the Al_2_O_3_ content increases, the sintering densification of the glass-ceramics was improved, whilst the crystallization was inhibited. The increase in sintering temperature reduces the viscosity of the glass and promotes crystallization, and small pores are easy to combine with adjacent pores to generate large pores, leading to the deterioration of the compactness of the glass-ceramics. This indicates that as the Al_2_O_3_ content increases, the crystallization of the glass is inhibited, and the sintering densification of the glass-ceramic is improved. RBAS glasses sintered at 850 °C exhibited irregularly shaped interconnected pores in the samples, indicating that glass crystallization has a certain inhibitory effect on sintering.

**Figure 10:**
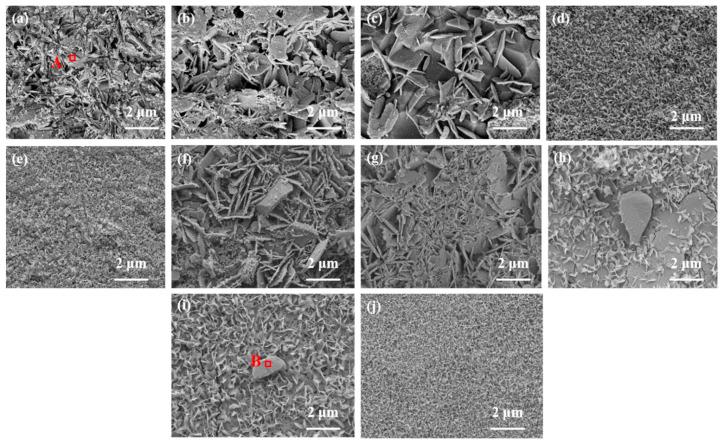
SEM images of RBAS basic glasses sintered at 800 °C or 850 °C after erosion by 5 wt% HF solution; (**a**–**e**): 800 °C B1–B5, (**f**–**j**): 850 °C B1–B5.

**Figure 11:**
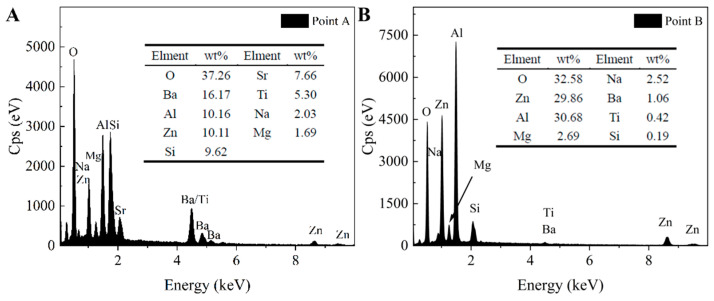
EDS of point A (**A**) and point B (**B**) of the RBAS glass-ceramics.

Images depicting the microstructure of RBAS glass-ceramics, which were sintered at either 800 °C or 850 °C and subsequently etched in a 5.00 wt% HF solution for 1 min, are presented in Figure 10. After RBAS glass-ceramic samples were etched, the crystalline morphology was exposed. The structure exhibited both plate-like and granular morphologies. The plate-like morphology dominated, indicating that BaAl_2_Si_2_O_8_ crystals are plate-like. For RBAS microcrystalline glass sintered at 800 °C, when the Al_2_O_3_ content is less than 17.5 mol%, as shown in Figure 10a–c, the plate-like morphology gradually increases in size with increasing Al_2_O_3_ content, from approximately 2 μm to 5 μm. When the Al_2_O_3_ content exceeds 17.5 mol%, as shown in Figure 10d,e, the plate-like morphology gradually decreases with increasing Al_2_O_3_ content. The plate-like morphology in Figure 10d has a size of approximately 1 μm, while in Figure 10e, it further decreases to about 500 nm. For RBAS basic glasses sintered at 850 °C, as the Al_2_O_3_ content increases, the content of plate-like morphology crystals decreases, and the morphology size decreases from approximately 5 μm to 800 nm. The results indicate that as the Al_2_O_3_ content increases, the precipitation of BaAl_2_Si_2_O_8_ crystals in RBAS glass-ceramics is suppressed, and the crystal size decreases. To further confirm the types of crystalline phases precipitated, regions with different morphologies were selected and subjected to energy-dispersive X-ray spectroscopy (EDS) testing. The locations selected for EDS testing were the plate-like morphology in Figure 10a and the granular morphology in Figure 10i. The EDS test results are shown in Figure 11. It can be seen that the main components of the EDS analysis of the plate-like morphology are O, Ba, Al, Zn, and Si, indicating that it is primarily BaAl_2_Si_2_O_8_ crystal. The main components of the EDS analysis of the granular morphology are O, Zn, and Al, suggesting that it corresponds to ZnAl_2_O_4_ crystal. The above analysis is consistent with the XRD results.

### 3.5. Property Analysis

**Figure 12:**
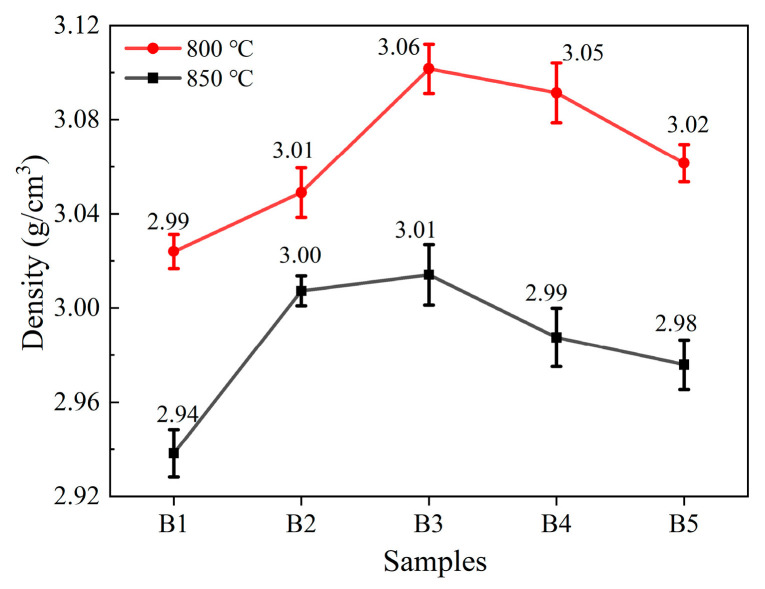
The bulk density of RBAS glasses after sintering at 800 °C and 850 °C for 10 min.

Figure 12 displays the bulk density of ABAS glass with varying Al_2_O_3_ contents after sintering at 800 °C and 850 °C for 10 min. As the Al_2_O_3_ content increases, the density of the glass-ceramics obtained after sintering at 800 °C and 850 °C initially increases and then declines, and the density of the glass sintered at 850 °C is lower than that sintered at 800 °C. This is because when the Al_2_O_3_ content increases from 12.5 mol% to 17.5 mol%, the sintering density of the glass increases, resulting in a more compact glass network structure, which leads to an increase in the volume density of the glass-ceramic. However, when the Al_2_O_3_ content continues to increase, the volume density decreases. When the Al_2_O_3_ content is between 20.0 mol% and 21.5 mol%, the significant reduction in crystallization also causes the density of the glass-ceramics to decrease. As demonstrated by the cross-sectional scanning electron microscopy analysis shown in Figure 9, an increase in sintering temperature promotes crystallization, resulting in pores forming where crystals precipitate, which causes the density of the glass-ceramics sintered at 850 °C to be lower than that of the glass-ceramics sintered at 800 °C.

**Figure 13:**
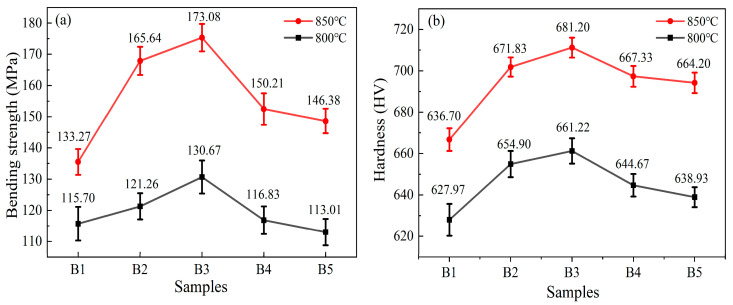
Flexural strength (**a**) and Vickers hardness (**b**) of the RBAS glass-ceramics sintered at 800 °C and 850 °C.

The bending strength and Vickers hardness of RBAS glass-ceramics are illustrated in Figure 13. The degree of polymerization of the glass, the type, and quantity of precipitated crystal phases are key indicators of the mechanical properties of glass ceramics [54]. As the Al_2_O_3_ content rises, the flexural strength and Vickers hardness of the RBAS glass-ceramics, which are sintered at either 800 °C or 850 °C, initially increase and subsequently decrease. As the Al_2_O_3_ content increases from 12.5 mol% to 17.5 mol%, the crystallinity declines slightly, yet the number of pores diminishes markedly, thus improving the mechanical properties. When the Al_2_O_3_ content falls within the range of 17.50 to 21.50 mol%, the crystallinity diminished substantially, and the number of pores decreased only slightly. The significant reduction in crystallization leads to a weakening of the mechanical properties. Furthermore, since an increase in the sintering temperature facilitates the precipitation of the crystal phase, the mechanical properties of the glass-ceramics sintered at 850 °C are superior to those sintered at 800 °C.

**Figure 14:**
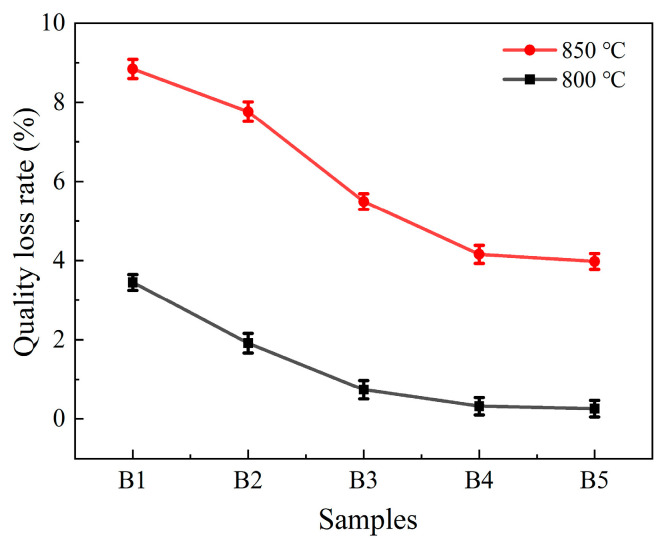
Quality loss of acid corrosion of the RBAS glass-ceramics sintered at 800 °C or 850 °C.

Figure 14 depicts the mass loss of RBAS glass-ceramics following their erosion in a 10 v% HCl solution for 30 min at 25 °C. It is observable that with the elevation of the Al_2_O_3_ content, the mass loss of the glass-ceramics, which has been sintered at either 800 °C or 850 °C and then subjected to HCl immersion, exhibits a downward trend. This can be attributed to two main factors: (1) The alkaline earth metal SrO demonstrates a pronounced tendency to react with the HCl solution. As the content of SrO diminishes, the mass loss is concomitantly mitigated; (2) The augmentation of the Al_2_O_3_ content stimulated the sintering densification process of the glass-ceramics, effectively reducing the mass loss. It is also notable that as the sintering temperature ascends, the densification degree of the glass-ceramics declines, resulting in greater mass loss at 850 °C compared to 800 °C. The acid resistance of glass ceramics is strongly associated with the microcrystalline phase, the glass phase, and the interface structure between the two phases. At the interface between the two phases, the atomic arrangement is relatively disordered, making it prone to defects [24]. These defects can serve as entry points for acid corrosion, accelerating the erosion of the material by acid and thereby reducing its acid resistance. Therefore, the acid resistance of glass-ceramics is not directly proportional to the content of the microcrystalline phase [55,56].

**Figure 15:**
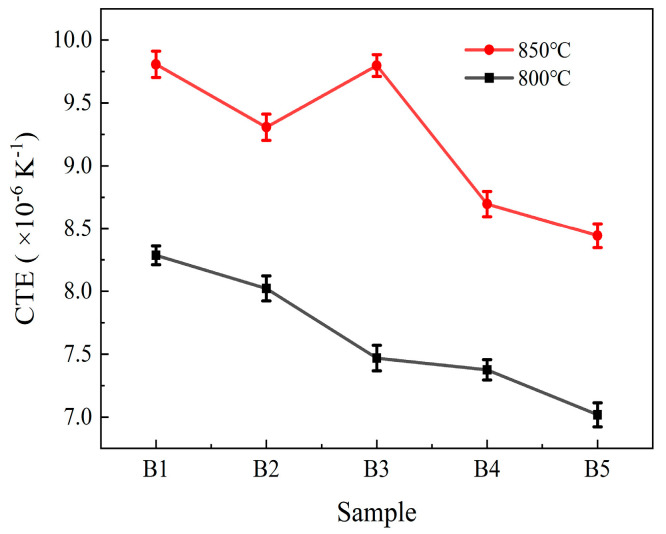
The CTE of the RBAS basic glasses sintered at 800 °C and 850 °C.

The CTE of RBAS glass-ceramics is depicted in Figure 15. As can be observed from Figure 15, regardless of whether the temperature is 800 °C or 850 °C (after sintering at 800 °C, the CTE range of Group B glass is 7.03–8.29 × 10^−6^ K^−1^; after sintering at 850 °C, its CTE is 7.23–8.58 × 10^−6^ K^−1^), the CTE of RBAS glass-ceramics decreases gradually with increasing Al_2_O_3_ content. This downward trend can primarily be rationalized by two main mechanisms: (1) The CTE of BaAl_2_Si_2_O_8_ crystals (8 × 10^−6^ K^−1^) and ZnAl_2_O_4_ crystals (7.7 × 10^−6^ K^−1^) are all higher than those of the glass phase [57]. As the Al_2_O_3_ content increases, the crystallinity of the sealed glass decreases, leading to a reduction in CTE. (2) As the Al_2_O_3_ content increases, the glass network polymerization of the glass-ceramics strengthens, further reducing the CTE. Moreover, elevated sintering temperatures promote crystallization, leading to an increase in the CTE of the glass-ceramics. From a review of the CTE range of Group B glasses, it can be observed that they match the CTE of Al_2_O_3_ ceramic, confirming their compatibility for low-temperature co-firing applications.

### 3.6. Glass-Ceramics Co-Sintered with Al_2_O_3_ Ceramic Substrate

**Figure 16:**
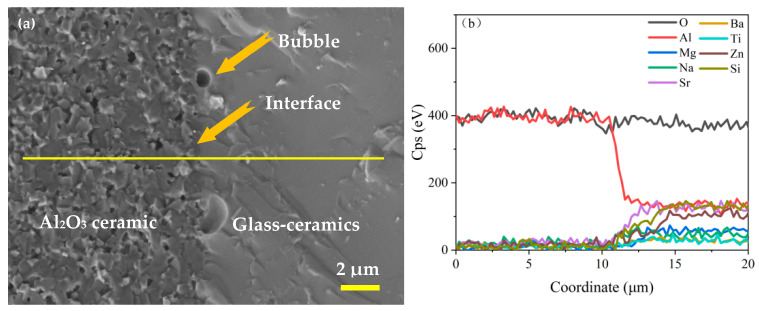
Interfacial morphology of B3 glass-ceramics and Al_2_O_3_ ceramic substrates co-sintered at 800 °C for 10 min.

Sample B3 exhibited the best overall performance when sintered at 800 °C and was therefore selected for co-sintering with the alumina ceramic substrate. Figure 16 shows the interface morphology (a) and elemental distribution (b) of B3 coated on an Al_2_O_3_ ceramic substrate and sintered at 800 °C for 10 min. The left portion of the image displays the morphology of the Al_2_O_3_ ceramic substrate, whereas the right portion presents the sealed glass, demonstrating favorable wettability and continuity. The yellow line marks the EDS scanning trajectory across the interface. The B3 glass forms a close bond with the Al_2_O_3_ ceramic substrate, with no interfacial cracks observed. Elemental distribution across the interface transitions gradually, accompanied by notable ion diffusion, leading to a secure adhesion between the glass and the ceramic substrate. These findings suggest that B3 glass-ceramics exhibit excellent chemical compatibility and a matching CTE with the Al_2_O_3_ ceramic substrate.

## 4. Conclusions

In this study, RBAS glass-ceramics were successfully prepared and applied to the packaging of Al_2_O_3_ ceramic substrate. The impact of Al_2_O_3_ content on the network structure and sintering properties of glasses were studied, and the following conclusions were reached:

(1) As the content of Al_2_O_3_ increases progressively, the degree of polymerization of the RBAS glass network exhibits a significant enhancement trend. Correspondingly, the T_g_, T_e_, and T_p_ of the glass-ceramics all rise accordingly. This series of changes in temperature parameters reflects the coordinated evolution of the internal structure and properties of the material.
(2) Upon the increment of Al_2_O_3_ content, a downward trend was observed in the overall crystallinity of RBAS glass-ceramics. Conversely, as the sintering temperature was elevated, the crystallization process of RBAS glass-ceramics was accelerated. However, this led to a degradation in the sintering densification degree.
(3) The RBAS glass-ceramics sintered at 800 °C with Al_2_O_3_ content of 17.50 mol% manifest the best comprehensive properties: bending strength of 130.58 MPa, Vickers hardness of 661.67 HV, and CTE of 7.45 × 10^−6^ K^−1^; the mass loss was 0.73%. The results show that the glass-ceramics had excellent chemical compatibility and a suitable CTE with Al_2_O_3_ ceramic substrates.

## Figures and Tables

**Table 1:** Chemical composition of the RBAS glasses/mol%.

Sample	SiO_2_	B_2_O_3_	Al_2_O_3_	SrO	ZnO	BaO	TiO_2_	MgO	Na_2_O	mol%
B1	29.00	16.00	12.50	9.00	9.50	8.00	6.00	6.00	4.00	100.00
B2	29.00	16.00	15.00	6.50	9.50	8.00	6.00	6.00	4.00	100.00
B3	29.00	16.00	17.50	4.00	9.50	8.00	6.00	6.00	4.00	100.00
B4	29.00	16.00	20.00	1.50	9.50	8.00	6.00	6.00	4.00	100.00
B5	29.00	16.00	21.50	0.00	9.50	8.00	6.00	6.00	4.00	100.00

**Table 2:** Chemical composition of the RBAS glasses tested by ICP-MS/mol%.

Sample	SiO_2_	B_2_O_3_	Al_2_O_3_	SrO	ZnO	BaO	TiO_2_	MgO	Na_2_O	mol%
B1	28.94	16.06	12.62	8.91	9.62	7.96	6.06	5.89	3.94	100.00
B2	28.84	15.95	15.28	6.41	9.7	8.06	5.96	5.95	3.85	100.00
B3	29.09	16.09	17.64	3.88	9.72	7.88	5.92	5.91	3.87	100.00
B4	28.73	15.97	20.36	1.43	9.6	8.11	6.11	5.87	3.82	100.00
B5	28.93	16.03	21.74	0	9.58	7.9	6.02	5.92	3.88	100.00

**Table 3:** The assignments of FTIR spectra of the prepared basic glasses.

Wavenumber (cm^−1^)	Assignments	References
450	Si–O–Si bending vibration in [SiO_4_]	[34]
710	B–O–B bending vibration absorption peak in [BO_3_], and Si–O–Al symmetric stretching vibration	[35,36]
800~1200	Si–O–Si antisymmetric stretching vibration peak in [SiO_4_], B–O–B antisymmetric stretching vibration peak in [BO_4_], and Ti–O vibration peak in [TiO_4_]	[37,38,39]
1260	B–O stretching vibration peak in [BO_4_]	[35,36]
1390	B–O–B antisymmetric stretching vibration peak in [BO_3_]	[35,38]

**Table 4:** The distributions of Raman spectra of the RBAS basic glasses.

Raman Shift (cm^−1^)	Raman Assignments	References
300	The vibration of cation	[40,41]
550	Al–O–Al	[42,43]
700~705	Ti–O vibration in [TiO_6_] octahedra	[44,45,46]
774~780	Ti–O vibration in [TiO_5_] tetragonal pyramid	[44,45,46]
859~862	Ti–O vibration in [TiO_4_] tetrahedron	[44,45]
908~910	Si–O–Al	[42,47]
933~936	Si–O– stretching vibration of [SiO_4_] (Q^1^)	[48,49]
980~988	Si–O– stretching vibration of [SiO_4_] (Q^2^)	[48,49]
1045~1050	Si–O– stretching vibration of [SiO_4_] (Q^3^)	[48,50]

**Table 5:** Area fraction of TiO_x_ and Q^n^ (Si) (%).

Sample	Ti (100%)			Si (100%)		
	[TiO_4_]	[TiO_5_]	[TiO_6_]	Q^1^	Q^2^	Q^3^
B1	64.17	22.87	12.96	20.30	39.44	40.26
B2	63.53	22.37	14.10	19.76	39.25	41.02
B3	62.91	22.00	15.12	17.91	38.78	43.31
B4	62.67	21.14	16.19	16.06	37.41	46.53
B5	61.72	20.58	17.71	14.19	36.69	49.12

**Table 6:** Characteristic temperatures of the basic glasses (°C).

	T_g_	T_e_	T_p1_	T_p2_	T_p3_
B1	591	729	-	-	813
B2	599	737	-	748	823
B3	602	750	703	775	827
B4	613	757	705	782	859
B5	619	774	712	798	872

## Data Availability

The original contributions presented in this study are included in the article. Further inquiries can be directed to the corresponding author.

## Abbreviations

The following abbreviations are used in this manuscript:

LTCC	Low-temperature co-fired ceramic
CTE	Coefficients of thermal expansion
CBS	CaO-B_2_O_3_-SiO_2_ glass
MBS	MgO-B_2_O_3_-SiO_2_ glass
ZBS	ZnO-B_2_O_3_-SiO_2_
LBA	Li_2_O-B_2_O_3_-Al_2_O_3_
LAS	Li_2_O-Al_2_O_3_-SiO_2_
RTBS	RO-TiO_2_-B_2_O_3_-SiO_2_
RBAS	SrO, BaO, ZnO, MgO (RO)-B_2_O_3_-Al_2_O_3_-SiO_2_
SOFC	Solid oxide fuel cells
ICP-MS	Inductively coupled plasma mass spectrometry
DSC	Differential scanning calorimetry
FTIR	Fourier infrared spectrometer
XRD	Diffraction of X-rays
FE-SEM	Field emission scanning electron microscopy
HTM	High-temperature microscope
